# Developing the Community Engaged Physician: Medical Students Reflect on a Household Visit Curriculum

**DOI:** 10.7759/cureus.11593

**Published:** 2020-11-20

**Authors:** Sarah Stumbar, Onelia Lage, Ebony B Whisenant, David R Brown

**Affiliations:** 1 Family Medicine, Herbert Wertheim College of Medicine, Miami, USA; 2 Pediatrics, Herbert Wertheim College of Medicine, Miami, USA

**Keywords:** medical education, social determinants of health, house calls, reflective practice, medical humanities, professional identity formation

## Abstract

Introduction: To educate students about social determinants of health, our medical school assigns interprofessional student teams to work longitudinally with underserved households to identify and address their health and social needs. To cultivate reflective practice--an essential component to training competent professionals through service-learning programs--students are asked to recognize their emotional responses to patient encounters. This project used reflective essays to identify the emotional responses of medical students to the start of their household visit experience and to assess their observations in relation to social determinants of health.

Methods: Thematic analysis was used to examine patterns in reflective essays provided by 99 medical students. Two independent reviewers read the essays and created initial codes, which were developed into a common codebook by consensus. Codes were categorized into themes, including observations of the social determinants and emotional reactions to household visits.

Results: Through the provision of household-centered care, medical students recognize the roles that social determinants play in the health of patients, households, and communities. Furthermore, they are able to identify household and community level interventions to address these identified needs. A variety of emotional responses to household visits were identified, ranging from frustration and sadness to empathy and humility.

Conclusions: Medical students undergo an emotional evolution even at the start of their household visit experience; highlighting that early patient care responsibilities play an important role in their development from pre-professional students to doctors-in-training. Additionally, student observations of the social determinants suggest that household visits can provide an opportunity for the application of knowledge about identifying and addressing these barriers to care.

## Introduction

For three decades, Healthy People has provided evolving evidence-based national objectives for improving health outcomes in the United States. While health disparities have been addressed by the Healthy People initiatives since Healthy People 2000, their focus on the social determinants of health has been progressively strengthened. First presented in 2010, the Healthy People 2020 Initiative recognizes that the health of individuals and populations is largely determined outside of the doctor’s exam room; constructed from the social and physical environments of patients. Healthy People 2020 identified five key social determinants: economic stability, education, social and community context, health and health care, and the neighborhood and built environment. The overarching goals of Healthy People 2020 focus on eliminating preventable disease, achieving health equity and eliminating disparities, creating healthy social and physical environments and promoting healthy behaviors [1].

As such, we must train future physicians who have a strong foundational understanding of the social determinants of health; and, more importantly, an understanding of how they might effectively use social services navigation and public and organizational policy to address the barriers faced by their patients. The American Association of Medical Colleges (AAMC) has recommended that medical schools teach population health and the behavioral and social sciences as a way to promote a greater understanding of the social determinants of health [2,3]. Furthermore, a 2016 report released by the Institute of Medicine (IOM) of the National Academies of Science, Engineering, and Medicine provided guidelines for training physicians capable of addressing the social determinants of health. The IOM report stated that “Educating health professionals about the social determinants of health generates awareness … about the potential root causes of ill health … contributing to more effective strategies for improving health and health care for underserved individuals, communities, and populations" [4].

Since its inaugural class entered the school in 2009, our institution has integrated teaching of the social determinants of health into the Neighborhood Health Education and Learning Program (NeighborhoodHELP). This program assigns medical students, as part of interprofessional teams, to a household in an underserved neighborhood in a major metropolitan area [5-8]. Under the supervision of interprofessional faculty, students, starting in their first year of training, provide care that addresses the medical and social needs of their assigned household. The backbone of this program is household-centered care; in this model, medical, nursing, and social work students provide holistic care in the home setting to members of a household. In this way, this program provides a community classroom for the practical application of ethical, social, behavioral, and clinical competencies.

NeighborhoodHELP also provides uninsured and underinsured patients with access to free medical care, social work and behavioral health services, an education and tutoring program, and a legal clinic. Students help their assigned household access and navigate needed services, with the understanding that addressing social determinants is an integral part of health care provision. This ultimately is a service-learning experience, defined by the Liaison Committee on Medical Education (LCME) as “a structured learning experience that combines community service with preparation and reflection" [9]. 

The purpose of this qualitative study was to better understand the education that our service-learning household visit program provides our students regarding the social determinants of health. We hypothesize that NeighborhoodHELP presents students with a more salient education regarding social determinants than what is taught in a traditional classroom, hospital, or office-based format. As such, we were looking to understand household visits as a means through which to expose medical students to real-world applications of social determinants. We sought to understand the students’ experiences through an analysis of their reflective essays written around the time of their first household visits. In these essays, students are asked to both describe their household visit experiences and to discuss their perceived significance. Multiple previous studies have set the precedent for using reflective essays to evaluate the specific experiences of students in the field [10-13].

In addition to describing student experiences related to the social determinants of health, this study included an analysis of students’ reports of their emotional responses to and reflections regarding their provision of household-centered care. As medical students develop their professional identity as physicians, they must be able to reflect on their own attitudes, beliefs, and values within the greater context of medical culture [14].

Regarding reflection, resilience, and relationships, Hedy Wald writes: “Key drivers of professional identity formation include experiential and reflective processes, guided reflection, formative feedback, use of personal narratives, integral role of relationships and role models, and candid discussion within a safe community of learners" [15]. To promote guided reflection following initial household visits, students participate in a reflections round session, where household visit experiences are discussed with a small group of students and a faculty member. Students then write guided reflective essays; these essays provide qualitative data for this study.

## Materials and methods

In September 2015, at the start of their second year of medical school and after the deadline for completion of their first household visit, students were required to participate in a two-hour Reflections Round session, which included five to eight students and a faculty member. Two sets of questions--one focused on the household visit experience and one on the interprofessional experience-were provided to help elicit discussion during these sessions. The prompts provided were as follows:

A. Have you ever been emotionally upset as related to your household?

B. What observations have you made of the house, community, and/or family interactions?

C. Can you think of any policy/advocacy ideas that can improve the quality of life of your household?

D. Has anything shocked you?

E. What has the household taught you?

F. How did you feel after you walked out of your first household visit?

Students were also asked to specifically reflect on their interprofessional experience working with students in nursing, law, and social work. They were provided with the following prompts:

A. What did you learn about the other professions (through your first household visit)?

B. How did you collaborate?

C. What was different about your communication style?

D. How did you address difficulties?

After participating in Reflection Rounds, students were required to write a two-page essay responding to one or more of the provided questions. Ninety-nine medical students from the class of 2018 consented to the inclusion of their reflective essays in this study. The essays of students who did not consent were not included in the pool of qualitative data. Institutional review board approval was received from our institution for qualitative analysis of the student reflective assignments.

Thematic analysis was used to examine patterns in qualitative data provided by the consented medical students’ essays [16]. Two independent physician reviewers read the data and created initial codes. The reviewers then met in person; and initial codes were further developed into a common codebook by consensus. Codes were categorized into themes, using quotations from essays to describe their meaning. The reflective essays were then reviewed again using the consensus codebook as a framework. Included in the analyses were student observations of social determinants of health and students’ emotional reactions to their first household visits.

## Results

Results of the thematic analysis of student reflective essays showed diverse observations about the impact of social determinants on their households. These observations included themes of the built environment, insurance status, lack of access to reliable transportation, immigration concerns, cultural and religious beliefs, and linguistic barriers.

Deep emotions were embedded in many of these descriptors of the social determinants of health. For example, in relation to the immigration experience of his household, one student writes, with much empathy: “I have heard about people who live in our country who are undocumented, but I have never known who they are until now. Throughout my time with my patient, I have realized that she has the same hopes and dreams as any American citizen.” Another student writes about his early experience of linguistic barriers with his household: “The patient used the phone interpreter to communicate with us, and at first was speaking so softly with his head down; we had to keep reminding him to speak up. About halfway through the visit, though, he began to speak more loudly, and smile ...”

Students had a diverse range of observations and experiences, citing social determinants that both positively and negatively impacted the health of their households. For example, the theme of the built environment includes students’ descriptors of both possible strengths and health barriers presented in the households’ communities. Many students also specifically commented on the particular socio-cultural strengths of their household’s experience of health and healthcare; this was particularly evident regarding observations about the supportive role that church plays in the lives of many of our household members. One student writes: “His evident devotion to his religion is one social determinant of health that we identified as a strength for this household.” The detailed codebook of identified themes related to the household visit experience and social determinants of health can be found in Table 1.

**Table 1:** Recurrent themes identified in student reflective essays, as they relate to their observations of the social determinants of health.

Identified Theme Related to Household Visits	Description of Relevant Social Determinants of Health	Representative Quotations from Reflective Essay
Communication with household	This includes difficulties contacting the household by phone, visit cancellations, and language barriers, including using an interpreter for the first time.	“Contact with my patient early on was few and far between; most of the time I found myself exchanging words with an answering machine.” "Later they mentioned that they felt embarrassed that they couldn't communicate with me more personally due to their discomfort with English; I reciprocated that I wish I knew more Spanish."
Transportation resources of the household	This includes modes of transportation available to the household (ie. personal car, rides from friends, or public transit), transportation difficulties experienced by the household, and the direct impact of transportation difficulties on the household’s health.	“They have stopped using public transportation because they can no longer afford it.” "...the family lives relatively close to the metro, which means that the patient can walk to the station ... although this introduces safety concerns in terms of coming home at night and crossing the street."
Insurance status of household	This includes the insurance status of all members of the household, including specific types of insurance (Medicaid, Medicare, ACA/”Obamacare”), inadequate insurance, uninsured status, and financial constraints on the household’s ability to adhere to recommended medical treatment.	“My household fell into the insurance gap … too much money for Medicaid but not enough to make subsidized insurance affordable.”
Legal concerns of household	This includes issues such as immigration and documentation concerns, citizenship, denial of government benefits, bankruptcy/foreclosure, hearings for disability benefits, and various other matters that require legal assistance.	“The main social issue she has been dealing with is that she is an undocumented resident.”
Physical environment of household and community	This includes safety hazards within the home, such as a lack of air-conditioning, the presence of mold, bars on windows, dilapidated houses and overgrown lawns/yards; crime/violence within the home and/or in the neighborhood; ease of access to community services such as grocery stores, parks and libraries; and proliferation of fast-food eateries in the neighborhood. Additionally, this includes positive descriptors of many households’ interiors, i.e., very clean, well-kept, and welcoming.	“The lack of a central cooling system was immediately noticeable…” “The neighborhood is run-down but lively … [with] homes with anti-burglary bars in their windows.” "... it was clear that they had put in the effort to make their home presentable ..."
Cultural background of household	This includes the cultural, religious, and personal beliefs of the household; support received by the household from church and/or community fellowship; the impact of religion/spirituality on the household’s acceptance of Western medicine, and the household’s commitment to volunteering through the church and/or community.	“His evident devotion to his religion is one social determinant of health that we identified as a strength for this household.” "The patient has strong beliefs in herbal medicine and grows many medicinal herbs in her garden …”
Medical concerns of household	This includes medical issues faced by the household; such as the management of obesity, hypertension, and diabetes. It also includes an emphasis on dietary management and counseling. The impact of hospitalizations on a household member may have also been discussed.	“…it became obvious that she didn’t know why she was taking many of the medications … some of the medications were for conditions that she didn’t even know that she had.” "Her husband slowly walking into the room ... I knew that his history was significant for hypotension; however, as he walked past us ... I realized how severe his hypotension was. He was very uneasy walking ..."

In their narrative essays, students also discussed their emotional experiences and reactions to working, in collaboration with their interprofessional team, to address the social determinants of health experienced by their household. Their emotional experiences of household visits were wide-ranging, from anxiety and frustration to empathy and humility. For example, one student writes: “After walking out of my first household visit last spring with the sun beating down and the sound of children playing far down the block, I was experiencing many different emotions, but most of all I felt humbled.” Another student writes with surprise and a feeling of accomplishment, “… I was shocked that our team was able to help our household in so many different ways. There is currently a referral for her mother’s immigration status … social work agreed to help her with her resume and to apply for … [various social service programs] … It shocked me to see how much I could help a household just by listening to her and being there emotionally. ...”

These were not only emotional reactions to the household visits themselves, but also to the households’ community environments, the healthcare system, and the interprofessional team experience. One student articulates the emotional evolution experienced by him and many of his peers: “I turned to the patient and asked her right out-‘how can we help you?’ It is such a simple question … I realized that I had been approaching my patient as I would a test question … trying to spot key elements that would lead me to my answer. But after asking this simple question, I suddenly felt different. I felt like a healthcare provider, not a student.” The detailed codebook of identified themes related to the emotional experience of students on their household visits can be found in Table 2.

**Table 2:** Emotional reactions and themes related to the household visit experience, as identified in student reflective essays.

Identified Emotion Related to Household Visits	Description of Identified Emotion	Representative Quotations from Reflective Essay
Frustration	This includes frustration with the household, i.e., not responding to phone calls or adhering to an advised treatment plan; frustration with the inter-professional team; and frustration with the circumstances affecting the household.	“The overt poverty that my family lives in made me frustrated.” “The biggest frustration for me was scheduling the first household visit … I encountered many initial difficulties.”
Gratitude	This includes gratitude toward the household for welcoming the student into their home, gratitude for opportunities the student has in the program, and the student’s self-reflective gratitude.	“We built such an amazing bond … as I was leaving her house, she said out loud ‘this is your home.’” "Every time I go on a visit, I feel very welcomed and that my suggestions are very well received. I have learned how appreciative patients are to be educated on these topics."
Sadness	This includes sadness and surprise about the social and economic difficulties affecting the household, as well as the student’s sadness over his/her inability to “fix” the household’s circumstances.	“It is devastating to see this family go through more than they were already facing.” “It’s been pretty tough seeing such a kind-hearted family go through something like this…” “I was absolutely stunned by the fact that Mr. S had $118 to spend on himself and his son every month … the house looked bare … the refrigerator was essentially empty.”
Inspiration	This includes the student’s inspiration to develop a close relationship with the household, to develop a strong professional relationship with the interprofessional team, and to develop care plans that best serve the household’s needs. This also includes being inspired by the strength of the household’s resilience in the face of adversity.	“I wanted to do what I could to help her improve her health and her home life.” "Despite all of these things, the head of this household exhibits a great sense of strength and confidence in her speech, countenance, and body language. ... Her big smile lights up the street...It is incredible to see." "I was shocked at how nice it was inside. The kitchen was clean, the living room had a flat-screen TV and there was even a basket of fresh fruit on the table.”
Accomplishment	This includes instances when the student team was able to connect the household with primary care, legal services, and other specialist services; as well as instances when a household member was able to adhere to care plans developed by the student team.	“I talked to her about weight loss and exercise and since then she has been adherent to all of the things I suggested … she has kept a food diary, has been exercising daily and checks her blood pressure twice daily and records it.”
Humility	This includes feeling humbled by the household’s positive attitude, warmth, and kindness; even in the face of difficult health problems or socioeconomic barriers.	“It is humbling to contrast their living situation with what I have been blessed to experience throughout my life.” "Observing her vulnerability yet acknowledging the strength in which she handled the situation made me realize that the measly complaints I had about everyday life were nothing in comparison."
Anxiety	This includes anxiety about contacting the household, the first household visit, and an ability to truly help the household.	“On that very first home visit, I felt petrified. What could a second year medical student provide to this family? Could I help?” "We were entering their space, this wasn't the typical office or hospital environment I was familiar with ... How should I sit on the couch? ... When offered tea, is refusing rude or is accepting imposing?"
Empathy	This includes instances where the student identifies with the household and/or sees a situation in the household from the household’s perspective.	“As I listened to Ms. S talk about how she wished she could do more for her family, I understood her feelings of helplessness and frustration.” “I put my arms around her as she wept and told her that we would continue to work as hard as we could to ensure the best possible outcome for her current situation.”

## Discussion

Student observations regarding social determinants began before the first NeighborhoodHELP household visit even occurred; with linguistic barriers becoming evident during early telephone calls to the household. As students made initial phone contact with their assigned households and then completed their first visits, they were able to contextualize their households within their built environment-both at the individual household and community level. They had robust observations about their households’ homes, streets, and communities. They commented on the lack of air conditioning, which left them dripping with sweat and dehydrated. They noted the wide array of community fast-food options, and how this might make it difficult for household members to find healthy produce and meals. They described deserted streets and bars on the windows of homes; and discussed how people may not feel safe to exercise outside in this environment. They noted how their household members had to walk through the same empty streets to get to the bus stop, and then wait for the bus; and how these transportations difficulties would make it challenging to get to doctor’s appointments.

Furthermore, student discussions of the built environment revealed an array of conflicting emotions. Sometimes students were shocked and saddened by the dilapidated state of a household’s home or community; but at other times, they expressed surprise at the home’s clean and welcoming environment. These early household visits challenged students’ pre-conceived ideas about the people who live in the neighborhoods that our program serves. Sometimes, this meant that they observed poverty and homes that were previously unimaginable to them. At other times, this meant that they were shocked by the similarities between themselves and their assigned household. Deep empathy for their households was often embedded in the recognition of these similarities.

Student observations of the built environment speak to the value of continuity clinical responsibility early in training. These observations-and their accompanying emotions-are not available in a more traditional classroom-based curriculum. The combination of observations related to the social determinants and the students’ emotional reactions as they processed these early clinical encounters allows not only for the accumulation of knowledge but also for the acquisition of the emotional skill needed to manage the realities of their patients.

As students reflected on their first household visits, they were able to identify and discuss myriad social determinants; these included the built environment, cultural differences and beliefs, literacy and linguistic barriers, immigration concerns and access to reliable transportation. They expressed inspiration, humility, and empathy when considering the barriers facing their households; as well as sadness that they could not adequately address all of their households’ needs. Regardless of the exact emotional descriptor, there was a common sense of responsibility and desire to ensure that their households were receiving the most comprehensive and appropriate care possible.

For many students, their household provides their first experience of caring for someone in the role of a medical professional. In their reflective essays, students describe a wide array of emotions--from frustration to empathy to accomplishment--as a sense of responsibility for their household grows. As these essays were written at the start of their program experience, they suggest that students begin an emotional evolution even as students are first being introduced to their household.

Much has been written about the process of medical students’ identity formation as they become physicians. The idea of situated learning emphasizes that the professional identity of a physician begins to form as students gradually take on more responsibility for the patient care within a clinical context [17]. For most students at our institution, their assigned household constitutes their first patient; and, as such, their first experience with legitimate participation in patient care. Many of the emotional themes identified in our analysis relate both to students simultaneously processing the observed social determinants of their households and reacting to their first patient care experiences.

The thematic analysis presented here is limited by the fact that the students’ reflective essays were written in response to multiple different prompts; and students either responded to one or to a variable combination of questions. This means that the recurrence of themes could not be quantified by importance, as students chose to respond to a different compilation of prompts. Furthermore, students were at different stages of their household visit experience. Most students had completed at least one visit prior to the assignment, but other students had only participated in the course didactics because they were experiencing challenges in coordinating a household visit. While this variation may be seen as a limitation, it also accurately represents service-learning programs in the variable experiences that they provide.

## Conclusions

This analysis provides information about social determinants as they are observed and addressed by medical students at the start of their NeighborhoodHELP household visit experience. Even as students completed their first visits, they were able to contextualize their households within their built environment; making robust observations about homes, streets, and communities. Student observations and experiences of the built environment speak to the value of hands-on service-learning programs that provide experiences not available in a traditional classroom or clinical settings. This suggests that a household visitation curriculum can provide relevant exposure and teaching about social determinants.

In their reflective essays, students describe a wide array of emotions as their sense of responsibility for their household grows. As these essays were written at the start of the household visit experience, they suggest that the emotional work of professional identity formation starts even during the first months of the program. Future studies will explore reflective essays across the three years that students participate in the program.
